# Pulmonary Ultrasound in Patients with Heart Failure - Systematic Review

**DOI:** 10.5935/abc.20180097

**Published:** 2018-07

**Authors:** Rafael Tostes Muniz, Evandro Tinoco Mesquita, Celso Vale Souza Junior, Wolney de Andrade Martins

**Affiliations:** 1 Programa de Pós-graduação em Ciências Cardiovasculares da Universidade Federal Fluminense (UFF), Niterói, RJ - Brazil; 2 Complexo Hospitalar de Niterói, Niterói, RJ - Brazil

**Keywords:** Heart Failure, Pulmonary Congestion, Extravascular Lung Water / diagnostic imaging, Lung / diagnostic imaging, Ultrassonography, Lung / radiography

## Abstract

Pulmonary congestion is an important clinical finding in patients with heart failure (HF). Physical examination and chest X-ray have limited accuracy in detecting congestion. Pulmonary ultrasound (PU) has been incorporated into clinical practice in the evaluation of pulmonary congestion. This paper aimed to perform a systematic review of the use of PU in patients with HF, in different scenarios. A search was performed in the MEDLINE and LILACS databases in February 2017 involving articles published between 2006 and 2016. We found 26 articles in the present review, 11 of which in the emergency setting and 7 in the outpatient setting, with diagnostic and prognosis defined value and poorly studied therapeutic value. PU increased accuracy by 90% as compared to physical examination and chest X-ray for the diagnosis of congestion, being more sensitive and precocious. The skill of the PU performer did not interfere with diagnostic accuracy. The presence of B-lines ≥ 15 correlated with high BNP values (≥ 500) and E/e' ratio ≥ 15, with prognostic impact in IC patients at hospital discharge and those followed up on an outpatient basis. In conclusion, when assessing pulmonary congestion in HF, PU has an incremental value in the diagnostic and prognostic approach in all scenarios studied.

## Introduction

Heart failure (HF) is one of the major causes of hospitalization of adults in Brazil. The BREATHE Registry is the first to include a large sample of hospitalized patients with decompensated HF of different regions from Brazil,^1^ that being the first cause of hospitalization of patients older than 65 years,^2^ one fourth of whom are readmitted to the hospital within 30 days.^3^ In Europe, 44% of the patients with HF are readmitted at least once every 12 months.^4^ Acute or progressive dyspnea due to pulmonary congestion is the major reason why patients seek care in emergency units.^5^ Subclinical congestion is associated with a worse clinical outcome.^3,4^

Physical examination and chest X-ray are widely used by emergency doctors; however, they have low accuracy to diagnose pulmonary congestion. In addition, chest X-ray often depends on the radiologist's assessment, which delays decision-making.^6^

Pulmonary ultrasound (PU) was previously considered of little clinical usefulness in classic cardiology textbooks.^5^ However, since the study by Daniel Lichtenstein in 1997,^6^ PU has become widely used to assess alveolar-interstitial syndrome, which encompasses pulmonary congestion of cardiac origin,^6^ in intensive care and emergency settings, for hospitalized patients before hospital discharge, and for patients with HF undergoing outpatient follow-up.

The major use of PU for the cardiologist is to assess B-lines.^7-9^ The analysis of B-lines (ultrasound lung comets) allows the detection of alveolar-interstitial syndrome and the access to extravascular lung water.^6,7^ The B-lines are laser-like vertical hyperechoic reverberation artifacts that arise from the pleural line, extend to the bottom of the screen without fading and move synchronously with lung sliding.^10^ Several B-lines are present in pulmonary congestion and can aid the detection, semiquantification and monitoring of extravascular lung water, the differential diagnosis of dyspnea and the prognostic stratification of chronic and acute HF.^6,11^ When three or more B-lines are identified, the zone or field is considered positive.^7,10,12^

**Figure 1:**
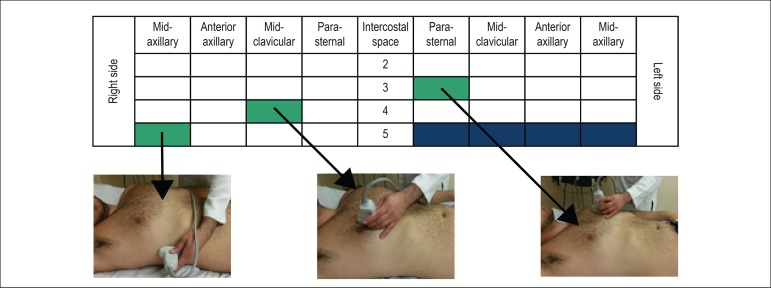
Methodology for pulmonary ultrasound assessment: 28 fields (zones). Modified from Jambrik et al.^15^

**Figure 2:**
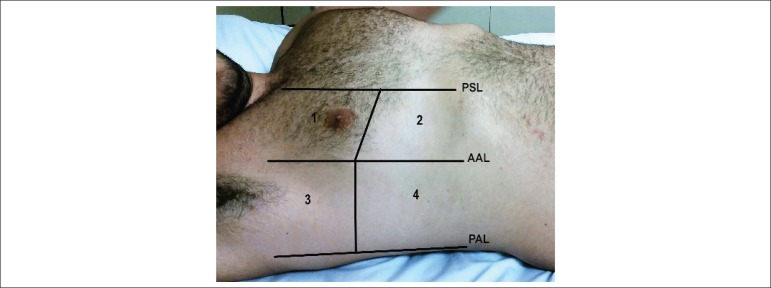
Methodology for pulmonary ultrasound assessment: 8 fields (zones). Modified from Volpicelli et al.^12^ PSL: para-sternal line; AAL: anterior axillary line; PAL: posterior axillary line.

Different methodologies have been applied to PU to analyze B-lines, from the prehospital setting, where only 2 lung fields are assessed,^13,14^ to more detailed assessments with 28 fields, as described by Jambrik^12,15^ (Figure 1). Most studies, however, have used the 8-field methodology as shown in Figure 2.

Pulmonary ultrasound has shown better accuracy than physical examination and lung X-ray for the diagnosis of pulmonary congestion, even when performed by physicians lacking training in the method or physicians other than radiologists.^16,17^ This method adds value to neuropeptides [brain natriuretic peptide (BNP) and NTpro-BNP] for the diagnosis,^18^ prognosis and treatment of patients with decompensated HF.

This study was aimed at conducting a systematic review about the use of PU for patients with HF in different clinical scenarios, to identify its role in the diagnosis, prognosis and treatment of the condition. We hypothesized that PU applied to the analysis of pulmonary congestion in different clinical scenarios for patients with HF can contribute to clinical practice.

## Methods

### Bibliographic search

The search was conducted in the MEDLINE (accessed via PubMed) and LILACS databases. The descriptors used were "heart failure", "pulmonary ultrasound", "thoracic ultrasound". The search in the databases used the following connectors: (heart failure) AND (pulmonary ultrasound) AND (thoracic ultrasound). The inclusion criteria adopted in the studies were: articles written in English, Portuguese or Spanish, approaching PU for the assessment of dyspnea or congestion in patients with HF. The data were extracted in a standardized way, by two independent researchers responsible for assessing the methodological quality of the manuscripts. Duplicate articles, reviews, editorials, letter to the editor, and studies conducted on animals and populations younger than 18 years were excluded. The search in the literature was performed in February 2017 and included articles from 2006 to 2016.

**Figure 3:**
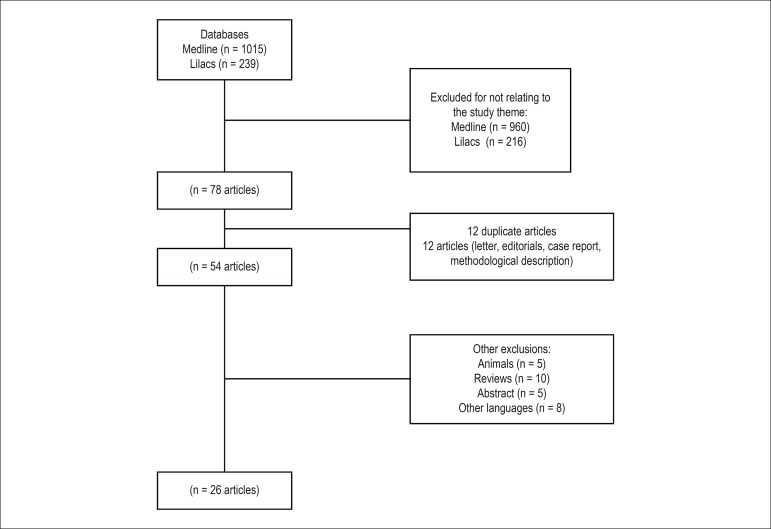
Structured search according to the PRISMA model of systematic reviews.

The articles were selected in two steps. In the first, the abstracts were read and those not meeting the inclusion criteria were excluded. In the second step, the studies selected based on their abstracts were fully read, and those not meeting the inclusion criteria were excluded, according to the PRISMA model (Figure 3).

## Results

### Interobserver assessment in pulmonary ultrasound and comparison with other diagnostic methods

Gustafsson et al.^19^ have observed that nurses specialized in HF and trained in PU for 4 hours achieved a substantial level of interobserver analysis when compared to cardiologists (k = 0.71 and 0.66) to assess B-lines and pleural effusion, respectively.^19^ Those results and other data are shown in Table 1.

**Table 1:** Summary of the articles selected and their results.

Diagnostic assessment of dyspnea in prehospital settings (AHF or DCHF)
PU was useful for the diagnosis in 68% of dyspneic patients in the prehospital setting with no delay in treatment and/or transportation, PE being present in 100% of those with decompensated HF, in 17% of patients with ACS, and in 20% of patients with COPD (p < 0.01), PE thus being a diagnostic marker in patients with decompensated HF.^13^ In the diagnosis of HF on PU, the S = 100% and E = 95% were comparable to those of NT-proBNP (> 1.000 pg/mL), S = 92% and E = 89%, and superior to those of the modified Boston criteria, S = 85% and E = 86%. The combination of PU and NT-proBNP showed S and E of 100%.^18^
**Diagnostic assessment of dyspnea in emergency settings (AHF or DCHF)**
Studies reported S ranging from 70% to 96.2% and E from 54% to 75%,^23-25,27,29,31^ diagnostic reclassification ranging from 19% to 47%,^23,24^ with change in treatment in 43% of the cases,^24^ figures comparable to those of BNP > 500 (S = 75% and E = 83%).^27^ PU accuracy of 90% versus 67% (p = 0.0001) for clinical examination, and 81% (p = 0.04) for the combination of clinical examination + NT-proBNP + X-ray.^25^ PU was better for the diagnosis of DCHF (S = 100%) and of PNM (S = 75%) as compared to stethoscope auscultation (S = 89% and S = 73%, respectively).^26^ Interobserver agreement was better in the anterior/superior thoracic zones for both pairs expert/expert and expert/beginner,^16^ and the PU performed by beginners versus experts had S and E of 79-85% and 84-88%, respectively,^17,37^ and PPV of 64-75% and NPV of 90.9-94%.^17,29^ Global agreement with the gold-standard method for pulmonary edema interpretation on PU was 74%, higher than that with X-ray (58%, p< 0.0001).^28^ A combination of PU and US of IVC had S = 94.3%, E = 91.9%, NPV = 91.9% and PPV = 94.3% to differentiate AHF from pulmonary disease,^29^ and JVD-US is a sensitive test (S = 98.2%) to identify pulmonary edema in dyspneic patients with suspicion of congestive AHF.^30^ Studies have shown an LR(+) of PU of 3.88-4.8% and an LR(-) of PU of 0.20-0.50%^24,31^ for the diagnosis of AHF or DCHF, being higher than the LR(+) of NT‑proBNP [= 2.3] and similar to the LR(-) of NT-proBNP [= 0.24].^31^
**Diagnostic assessment in intensive care settings (AHF or DCHF)**
Agreement of PU with the final diagnosis was 84%, with S = 86% and E = 87% for cardiac pulmonary edema,^32^ and IVC values > 9 mm on B mode had S = 84.4% and E = 92.9% [LR(+) = 11.8, LR(-) = 0.16] for the diagnosis of cardiac dyspnea.^33^
**Diagnostic assessment in outpatient settings**
Primary outcome (hospitalization due to DCHF and all-cause death) was 4x more frequent in patients of the third tertile than in patients of the first tertile with B-lines ≥ 3 (p < 0.001), whose time alive or outside the hospital was shorter (p< 0.001).^36^ The finding of B-lines or PE or both increased the risk of death or hospitalization (p< 0.05)^19^ and correlated in a paired way with the estimates of PCWP (p < 0.001) and with the fluid impedance index (p < 0.001); the impedance monitoring alert detected clinical deterioration of HF with S = 92%, while B-lines ≥ 5 showed S = 83%.^35^ HF decompensation was present in 68% of the patients when the number of B-lines ≥ 15, and correlated with NT-proBNP > 1000 (p < 0.0001) and with an E/e’ ratio > 15 (p < 0.0001).^34^
**Prognostic assessment**
Event-free survival (all-cause death and re-hospitalization) of patients with HF and B-lines ≥ 30 was shorter than that of patients with B-lines < 30 (p < 0.0001) in 3 months^10^ and of patients with B-lines ≥ 15 in 6 months,^11^ and the presence of B-lines ≥ 30 was a predictor of death with BNP > 700 (p = 0.002).^10^
**Therapeutic assessment**
The number of B-lines reduced with treatment (p < 0.05), and the PU score showed a linear correlation with the radiologic (p < 0.05) and clinical scores (p < 0.05) and with BNP levels (p < 0.05).^8^
**Assessment of PU as compared to other diagnostic methods**
An increase in the number of B-lines correlated with LVEDV (p = 0.036);^20^ LV end-systolic diameter (p = 0.026);^20^ PW (p = 0.009);^20^ LV mass index (p = 0.001);^20^ RA volume index (p = 0.005);^20^ TR velocity (p = 0.005);^20^ measures of RA, DPAP, MPAP, PVR, all p < 0,005,^21^ and SPAP (p = 0.003-0,005),^20-21^ and, for each B-line, there was an increase of 1 mm Hg in SPAP and of 0.1 Woods units in RVP.^21^ In the analysis of the number of B-lines, the US device types used did not statistically differ (4 or 8 zones assessed; p= 0.67),^22^ but the clip duration did differ: 4 versus 2 seconds (p < 0.001 for 4 and 8 zones) and 6 versus 4 seconds (p = 0.057 for 4 zones; and p = 0.018 for 8 zones).^22^

AHF: acute heart failure; DCHF: decompensated chronic heart failure; HF: heart failure; PU: pulmonary ultrasound; COPD: chronic obstructive pulmonary disease; PE: pleural effusion; ACS: acute coronary syndrome; S: sensitivity; E: specificity; NPV: negative predictive value; PPV: positive predictive value; NT-proBNP: N-terminal pro-brain natriuretic peptide; LR(+): positive likelihood ratio; LR(-): negative likelihood ratio; US: ultrasound; X-ray: chest X-ray; PNM: pneumonia; IVC: inferior vena cava; JVD-US: jugular vein distension on ultrasound; PCWP: pulmonary capillary wedge pressure; BNP: brain natriuretic peptide; LVEDV: left ventricular end-diastolic volume; PW: posterior wall; LV: left ventricular; LA: left atrium; TR: tricuspid regurgitation; RA: right atrium; DPAP: diastolic pulmonary artery pressure; MPAP: mean pulmonary artery pressure; PVR: pulmonary vascular resistance; SPAP: systolic pulmonary artery pressure.

Platz et al.,^20^ assessing the B-lines with Doppler echocardiographic data, have found a correlation with left ventricular (LV) end-diastolic diameter (EDD - p = 0.036) and LV end-systolic diameter (p = 0.026), with septal wall thickening (p = 0.009), LV mass index (p = 0.001), left atrial volume index (p = 0.005), tricuspid valve regurgitation velocity (p = 0.005) and systolic pulmonary artery pressure (SPAP, p = 0.003).

In two distinct studies, Platz et al.^21,22^ have concluded that the clip duration is more important than the type of device used to analyze B-lines, and that the number of B-lines correlate with right atrial pressures, diastolic and systolic pulmonary artery pressures and central venous pressure, but correlated with neither pulmonary artery occlusion pressure nor cardiac index.

In our initial experience, pulmonary congestion detected on PU correlated better with SPAP than with EDD, 86% and 58%, respectively.

### Pulmonary ultrasound and diagnostic assessment

A study has identified pleural effusion in 100% of the patients with decompensated HF in the prehospital setting,^13^ and another by Prosen et al.^18^ has concluded that PU can differentiate cardiac from pulmonary dyspnea, mainly when associating with the use of BNP, observing an increase in diagnostic sensitivity and specificity for the association of PU and BNP.

In the emergency setting, Pivetta et al.^23^ have observed an increase in diagnostic accuracy, with reclassification of the diagnosis in 19% of the patients after PU. Russel et al.^24^ have found a change in treatment in the acute phase of around 47% of the cases. Gallard et al.^25^ have reported an accuracy of 90% when PU was compared to the clinical examination (67%, p = 0.001), as well as compared to the combination of clinical examination with NT-proBNP and chest X-ray (81%, p = 0.04). Oskan et al.,^26^ when comparing the diagnostic performance of PU and auscultation for the diagnosis of decompensated HF and pneumonia, have found sensitivity of 100% and 89% vs. 75% and 73%, respectively. Gullet et al.^16^ and Chiem et al.^17^ have found agreement between the little or newly trained observer and the highly trained observer in the interobserver analysis for the diagnosis of patients with dyspnea in the emergency setting. Regarding the diagnosis of decompensated HF in patients with dyspnea in the emergency setting, Anderson et al.^27^ have found similar values for PU (S = 70%) and BNP > 500 pg/mL (S = 75%). Martindale et al.^28^ have reported the superiority of PU (74%) *versus* chest X-ray (58%) in the global agreement with the gold-standard method for the diagnosis of pulmonary edema. Kajimoto et al.^29^ have reported that inferior vena cava (IVC) ultrasound associated with PU increases diagnostic sensitivity in acute HF *versus* primary pulmonary disease. Jang et al.^30^ have reported that the longitudinal and cross-sectional measures of the internal jugular vein at the end of exhalation is a sensitive test to identify pulmonary edema on chest X-ray in patients with suspected HF. Liteplo et al.^31^ have reported the superiority of PU as compared to NT-proBNP to differentiate chronic HF from chronic obstructive pulmonary disease with a positive likelihood ratio (LR)(+) of 3.88 (99% CI = 1.55 - 9.73), while NT-proBNP had a LR(+) of 2.3 (95% CI = 1.41 - 3.76).

In the intensive care setting, Dexheimer Neto et al.,^32^ using the BLUE protocol in dyspneic patients, have found an 84% agreement between PU and the final diagnosis of pneumonia or acute pulmonary edema (total kappa = 0.81). Yamanoglu et al.^33^ have detected the cardiac origin of dyspnea by using the caval index (sensitivity= 84.4% and specificity= 92.9%).

In our clinical practice, we observed that PU increases the diagnostic accuracy of pulmonary congestion, being better than the stethoscope auscultation in both the emergency and the cardiac intensive care unit settings.

In the outpatient care setting, Miglioranza et al.^34^ have reported that a number of B-lines ≥15 correlates with NT-proBNP > 1000 (p < 0.001), E/e' ratio >15 (p = 0.001) and clinical assessment (p < 0.001), with sensitivity of 85% and specificity of 83%, for the risk of decompensated HF. Maines et al.^35^ have reported a correlation between the presence of B-lines and the impedance fluid index (p < 0.001) of patients with HF at regular outpatient follow-up.

### Pulmonary ultrasound and prognostic assessment

In the outpatient clinic context, Platz et al.^36^ have identified that patients with more than three B-lines had a four-fold increase in the chance of hospitalization due to HF or of all-cause death, being worth noting that 81% of those patients had no compatible alteration in lung auscultation. Gustafsson et al.,^37^ studying 104 patients, have identified that the presence of B-lines or pleural effusion or both correlated with the increased risk of death or hospitalization (HR: 3-4; p < 0.05). In 2015, Gargani et al.^9^ and Corio et al.^10^ found prognostic value on hospital discharge for the number of B-lines ≥ 30 and ≥ 15, respectively, for all-cause death or event-free hospitalization in 3 and 6 months (p < 0.001 for both).

We found a mean number of B-lines of 12.2 ± 7.3 on hospital discharge. Five patients were hospitalized again in 90 days, with an event-free mean of 63.6 ± 25.7 days and a mean BNP value of 450.10 ± 409.96 pg/mL.

### Pulmonary ultrasound and therapeutic assessment

Volpicelli et al.^8^ have concluded that B-line pattern mostly clears after medical treatment and correlates with other parameters, such as radiologic (p < 0.05) and clinical (p < 0.05) scores of congestion and BNP levels (p < 0.05).

## Discussion

This systematic review was aimed at identifying scientific evidence about PU in HF. The results showed it increases the HF diagnosis accuracy in the prehospital and hospital settings with incremental prognostic value on the discharge of patients with decompensated HF and might play a role in guiding the treatment of patients with HF.

**Figure 4:**
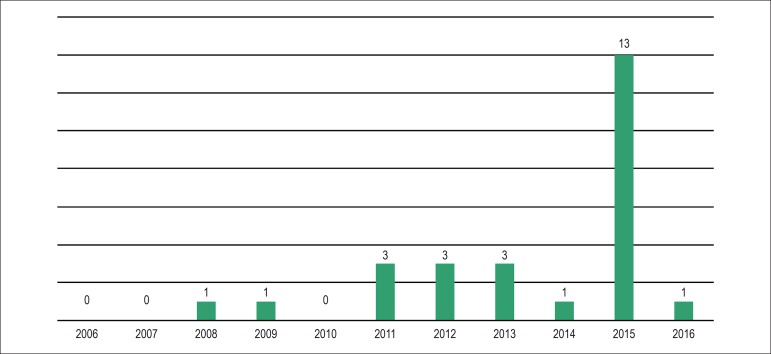
Distribution of specific publications about pulmonary ultrasound in heart failure in the 2006-2016 period.

Figure 4 shows the progressive increase in the number of publications on PU in HF over the past 10 years; however, several studies were clinical reviews,^7,38,39^ others were editorials, and there was a methodological description.^40^

**Figure 5:**
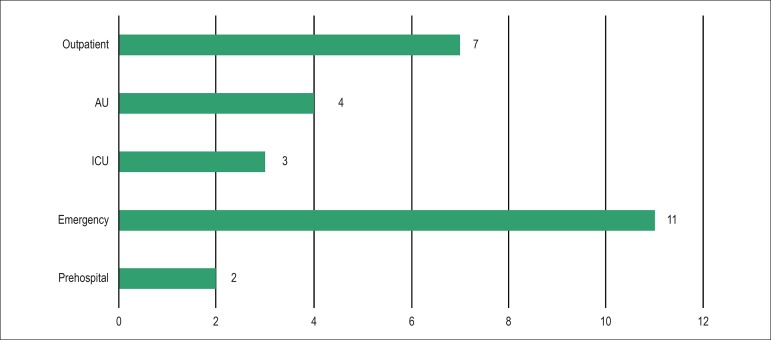
Distribution of the number of publications about pulmonary ultrasound in heart failure according to the assessment setting. AU: admission unit; ICU: intensive care unit.

There are several scenarios for the applicability of PU in assessing dyspneic patients with decompensated or presumed HF. As shown in Figure 5, the emergency application of PU was the most studied. It is believed that one of the reasons for that would be the low accuracy of physical examination and of chest X-ray^6^ for a rapid and more accurate diagnosis.^23,24^ A review study with 100 patients in the emergency department and using a pocket-sized cardiac ultrasound device has shown that PU can rapidly aid the diagnosis of HF, providing a more adequate and early treatment.^38^

In that context of emergency assessment, Miglioranza et al.^34^ and Facchini et al.^41^ have reported positive correlations between PU data and neuropeptide levels. That information can be useful, mainly when the measurement of natriuretic peptides is not available for the initial assessment. Another author,^42^ using PU in the emergency setting, has reported that the identification of multiple B-lines bilaterally was a sensitive, but not specific, predictor of BNP elevation > 500 pg/mL. That was the first study correlating B-lines with BNP.^42^ In addition, it was confirmed that the presence of alveolar-interstitial syndrome, identified by the presence of B-lines, can represent a precise and reproducible test to discriminate between cardiac and noncardiac dyspnea in the emergency setting, with sensitivity of 93.6%, specificity of 84%, positive predictive value of 87.9% and negative predictive value of 91.3%.^43^ Those findings also correlate with the NYHA functional class, left ventricular ejection fraction and grade of diastolic dysfunction.^44^

Several studies^5,18,23,24^ have correlated the presence of B-lines on PU with a sensitive marker for the diagnosis of decompensated HF; however, B-lines are not an exclusivity of decompensated HF. They can appear in adult respiratory distress syndrome and pulmonary interstitial fibrosis.^12^

Another review study of patients with HF followed up on an outpatient basis has concluded that PU has great diagnostic potential for identifying pulmonary congestion signs at the bedside, can become a state-of-the-art marker of interstitial fluid, and that the B-line pattern usually disappears after proper treatment of acute HF, revealing itself as an alternative diagnostic tool of easy use and therapeutic applicability.^8^ A recent systematic review has shown that the PU findings can rapidly change with therapy for HF, and that the identification of residual congestion in patients with acute HF at hospital discharge or in patients with chronic HF followed up on an outpatient basis can indicate those at higher risk for adverse events.^45^

Gullet et al.^16^ and Bedetti et al.^46^ have reported the excellent correlation between two observers with different specific expertise regarding PU for the analysis of B-lines at the bedside of patients with known or presumed HF.

In a study on stable patients undergoing dialysis, the identification of B-lines on PU correlated with pre-dialysis diastolic blood pressure (p = 0.015) and with the combination of reduced ejection fraction and reduced blood volume percentage at the end of hemodialysis (p = 0.028).^47^

We trained two non-specialized physicians on PU to assess congestion. We concluded that 4 hours of theoretical training and performing 15 tests were sufficient for them to develop similar accuracy in quantifying pulmonary congestion. Our tests are validated by a specialist radiologist (AMB), emphasizing our commitment with performance areas and need for proficiency-training.

In addition, in our medical practice, we identified the superiority of PU over stethoscope auscultation to assess pulmonary congestion. Furthermore, the presence of B-lines (mean value of 12.2 ± 7.3) was a marker of re-admission for one fourth of the patients in 90 days, and the presence of moderate congestion was a predictor of re-admission in 100% of the cases.

### Pulmonary ultrasound and evidence-based recommendations

Volpicelli et al.^12^ have proposed the first document to provide evidence-based recommendations for clinical use of point-of-care PU. In that document, those authors have determined the levels of evidence for each applicability, establishing that, when assessing interstitial syndrome, the ultrasonographic technique consists ideally of the assessment of 8 regions (range: from 2 to 28). A positive region is defined by the presence of at least three B-lines on a longitudinal plane between two ribs.

The ultrasonographic definition of B-line and the positive zone criterion (presence of ≥ 3 B-lines per field analyzed) were criteria used by all the authors of the present review. In addition, the criterion to define alveolar-interstitial syndrome (≥ 3 B-lines per field analyzed bilaterally) was common among the authors.

### Limitations

The present systematic review had as limitation the small sample size. The lack of standardization of the scores used for semiquantitative analysis was also a limiting factor.

## Conclusion

The use of PU to assess dyspneic patients and those with HF in different clinical settings increases the sensitivity, specificity and accuracy of the diagnosis and prognosis of pulmonary congestion in patients with HF.

Pulmonary ultrasound adds value to the diagnosis, facilitating decision-making in the assessment of acutely dyspneic patients, to whom HF is one of the differential diagnoses, minimizing treatment errors and improving the clinical outcome of this patient model.
